# Adiponectin Receptor Agonist AdipoRon Inhibits Proliferation and Drives Glycolytic Dependence in Non-Small-Cell Lung Cancer Cells

**DOI:** 10.3390/cancers16152633

**Published:** 2024-07-24

**Authors:** Sanober Kafeel, Angela Ragone, Alessia Salzillo, Giuseppina Palmiero, Silvio Naviglio, Luigi Sapio

**Affiliations:** Department of Precision Medicine, University of Campania “Luigi Vanvitelli”, 80138 Naples, Italy; sanober.kafeel@unicampania.it (S.K.); angela.ragone@unicampania.it (A.R.); alessia.salzillo@unicampania.it (A.S.); giuseppina.palmiero3@studenti.unicampania.it (G.P.); luigi.sapio@unicampania.it (L.S.)

**Keywords:** NSCLC, AdipoRon, glycolysis, AMPK

## Abstract

**Simple Summary:**

NSCLC is one of the most life-threatening forms of oncological diseases. Although targeted and immunotherapy treatments have improved NSCLC prognosis, the chance of surviving is still limited for many patients. Therefore, further efforts are required to improve NSCLC care. AdipoRon is emerging as an antineoplastic molecule in the treatment of different cancers, but its potential in NSCLC is yet to be explored. Herein, we demonstrated that AdipoRon strongly impairs viability, growth and stemness in NSCLC cells. We also recorded higher glucose consumption and lactate accumulation as a result of AdipoRon treatment. Remarkably, the employment of glycolytic-interfering agents enhanced its antiproliferative features. A signaling pathways analysis revealed a marked AMPK phosphorylation, while, in contrast, its abrogation by Compound-C significantly counteracted AdipoRon effectiveness. Altogether, these findings emphasize AdipoRon’s anticancer feature even in NSCLC, supporting its endorsement as a future candidate in cancer and NSCLC therapy.

**Abstract:**

Despite the countless therapeutic advances achieved over the years, non-small-cell lung cancer (NSCLC) is the leading cause of cancer-related death worldwide. To this primacy contribute both non-oncogene addicted and advanced NSCLCs, in which conventional therapies are only partially effective. The adiponectin receptor agonist AdipoRon has revealed antiproliferative action in different cancers, including osteosarcoma and pancreatic cancer. Herein, we investigated its potential anticancer role in NSCLC for the first time. We proved that AdipoRon strongly inhibits viability, growth and colony formation in H1299 and A549 NSCLC cells, mainly through a slowdown in cell cycle progression. Along with the biological behaviors, a metabolic switching was observed after AdipoRon administration in NSCLC cells, consisting of higher glucose consumption and lactate accumulation. Remarkably, both 2-Deoxy Glucose and Oxamate glycolytic-interfering agents greatly enhanced AdipoRon’s antiproliferative features. As a master regulator of cell metabolism, AMP-activated protein kinase (AMPK) was activated by AdipoRon. Notably, the ablation of AdipoRon-induced AMPK phosphorylation by Compound-C significantly counteracted its effectiveness. However, the engagement of other pathways should be investigated afterwards. With a focus on NSCLC, our findings further support the ability of AdipoRon in acting as an anticancer molecule, driving its endorsement as a future candidate in NSCLC therapy.

## 1. Introduction

According to the recent report issued by Cancer Statistics 2023, lung cancer represents the second-most predominant heterogeneous malignancy with a 12% rate of incidence, being the foremost cause of 21% of estimated deaths worldwide [1]. Non-small-cell lung cancer (NSCLC) is the most prevalent form of primary tumor affecting this parenchyma, while small-cell lung cancer and carcinoid tumors are, respectively, less common in the overall population [2]. NSCLC is typically classified into three major subtypes on the basis of their histological discrimination, namely lung adenocarcinoma (LUAD), squamous-cell carcinoma (LUSH) and large-cell carcinoma (LCLC) [3,4].

The pathogenesis of NSCLC often involves the deregulation of several signaling cascades affecting apoptosis, proliferation and growth [5,6]. As a result of progressive accumulation in both genetic and epigenetic abnormalities, the activation of multiple pathways confers growth advantage and positive selection during cancer evolution [7]. This phenomenon, termed as oncogene addiction, has been detected in all NSCLC subtypes, even though selectively targetable mutations often occur in young, non-smokers and female LUAD patients [8]. Several driver mutations have been reported in NSCLC, and more than fifteen related targeted agents have been approved by the Food and Drug Administration over the past few years [9]. Along with the targeted therapy, immune-checkpoint inhibitors (ICIs) have further revolutionized the clinical management of patients suffering from NSCLC [10,11]. However, as occurred with conventional therapies, limitations and weaknesses have also been experienced as a result of both targeted and immune therapies [12,13,14,15]. 

The present lack of adequate treatments leads the NSCLC prognosis to remain distressingly poor with a low 5-year survival rate [16]. Therefore, there is still an urgent need for alternative and well-tolerated therapeutic approaches for NSCLC.

AdipoRon is a newly synthetic molecule that acts as an agonist by stimulating the physiological responses of two well-known adiponectin receptors, ADIPOR1 and ADIPOR2 [17]. Several biological features have been linked to AdipoRon administration, such as anti-diabetic, anti-ischemic, anti-obesity and anti-depressant effects [18]. More recently, AdipoRon has also been proposed as an effective compound in suppressing cell growth in different cancer models, including myeloma, ovarian and prostate cancer [19].

We also provided evidence supporting the AdipoRon-mediated anticancer role in our earlier study, demonstrating that it affects cell cycle progression and inhibits the proliferation of Saos-2 and U2OS human osteosarcoma cell lines [20]. It has been shown that pancreatic adenocarcinoma (PDAC) represents the malignant disorder in which this compound has extensively been characterized. Akimoto and Messaggio were the first to corroborate AdipoRon’s efficacy in suppressing PDAC growth [21,22]. In line with the abovementioned findings, we recently investigated the pharmacodynamic interaction between AdipoRon and Gemcitabine, proving that their simultaneous administration revealed a striking action in contrasting PDAC growth and stemness than single ones [23]. Different signaling pathways have been reported to be modulated using AdipoRon, irrespective of the tumor type, such as mitogen-activated protein kinases (p42/p44 MAPK), AMPK and the mammalian target of rapamycin (mTOR) [20,21,23].

Although there are no ongoing clinical trials, AdipoRon has revealed high tolerability in animal models [24,25,26]. Salmons et al. have recently examined the safety of intravenous AdipoRon administration in a surgically stressed New Zealand White (NZW) rabbit model of arthrofibrosis [24]. All tested formulations of AdipoRon featured physiological pH ranges and a safe profile. Analogously, long-term AdipoRon treatment has been observed to promote ‘healthy ageing’ in obese middle-aged mice by enhancing endurance and protecting the skeletal muscle and liver against metabolic and degenerative effects [25]. Yamashita and colleagues reported no signs of toxicity after the prolonged administration of AdipoRon in a murine model of systemic sclerosis [26].

This current study examined the NSCLC responsiveness to AdipoRon for the first time, by addressing its anticancer potential on cell viability, growth and stemness. The metabolic state and signaling pathway were also investigated after AdipoRon treatment in the effort to provide a more comprehensive mechanism of action.

## 2. Materials and Methods

### 2.1. ADIPOR1 and ADIPOR2 Expression Analysis in NSCLC Patients and Cells

*ADIPOR1* and *ADIPOR2* expression levels of NSCLC patients were obtained from the Human Protein Atlas (HPA) program, using The Cancer Genome Atlas (TCGA) datasets [27,28]. Quantified raw sequencing data were downloaded in the available FPKM format (Fragments Per Kilobase Million). Based on the FPKM value of both genes, we divided NSCLC patients into two groups and examined their prognoses using Kaplan–Meier survival curves. To choose the best cut-off, all FPKM ranging from the 20th to 80th percentiles were investigated and the value yielding the lowest log-rank *p*-value was depicted on graphs. The expression and copy number levels of both ADIPOR1 and ADIPOR2 were gained in NSCLC cell lines by querying the Cancer Dependency Portal (DepMap) [29]. As reported in the interactive database, RNA-seq values were inferred using RNA-seq by Expectation-Maximization (RSEM) tool and stated in log2 transformation. Depending on the availability of the data type, log2-transformed copy number values were gathered from whole-genome sequencing (WGS), whole-exome sequencing (WES) or SNP array. Dot-plot charts were created combing mRNA and copy number results either by ADIPOR1 or ADIPOR2.

### 2.2. Chemicals and Antibodies

RPMI medium (ECB9006L; EuroClone, Pero, Italy), DMEM media (ECM0728L; EuroClone), AdipoRon (SML0998; Sigma-Aldrich, St. Louis, MO, USA), InSolution^TM^ AMPK Inhibitor, Compound-C (EMD Millipore Corp., Burlington, MA, USA), 2-Deoxy-D-glucose (2-DG) (D8375; Sigma-Aldrich), Oxamate (194131; Sigma-Aldrich), Ethanol absolute anhydrous (308603; Carlo Erba, Cornaredo, Italy), MTT solution (M2128; Sigma-Aldrich), Tween 20 (TC287; HIMEDIA, Mumbai, India), Phosphate-buffer solution (ECB4004L; EuroClone), Propidium Iodide (#P4864; Sigma-Aldrich), Penicillin-Streptomycin Solution 100X (ECB3001D; EuroClone), RIPA buffer (R0278; Sigma-Aldrich), Protease/Phosphatase Inhibitor Cocktail (#5872; Cell Signaling Technology, Danvers, MA, USA), Trypsin-EDTA 1X in PBS (ECB3052D; EuroClone), 2X Laemmli (S3401; Sigma-Aldrich), Ponceau staining solution (A2935; PanReac AppliChem, Castellar del Vallès, Spain), Fetal Bovine Serum (FBS) (ECS5000L; EuroClone), crystal violet (#C0775; Sigma-Aldrich), Dimethyl sulfoxide (DMSO) (A3672; PanReac AppliChem), Non-fat dry milk (A0530; PanReac AppliChem), L-Glutamine 100X (ECB3000D; EuroClone), Nitrocellulose Membrane (Amersham^TM^ Protran^TM^ Premium 0.45 µm, Sigma-Aldrich) and Excellent-Chemiluminescent Substrate Kit (E-IR-R301; Elabsciences, Houston, TX, USA) were all used in this study. The antibodies were as follows: AMPKα (#2532; Cell Signaling Technology), phopsho-AMPKα (#2535; Cell Signaling Technology), Antirabbit IgG HRP-linked Antibody (#7074; Cell Signaling Technology) and Vinculin (#13901; Cell Signaling Technology).

### 2.3. Maintenance of Cell Culture

H1299 and A549 NSCLC cell lines were obtained from the American Type Culture Collection (ATCC) and employed for experimental purpose in this study. H1299 cells were sustained in Roswell Park Memorial Institute (RPMI) medium along with the addition of L-Glutamine, while A549 cells were cultured in Dulbecco’s Minimum Essential Medium (DMEM). Both mediums were primed with 1% penicillin/streptomycin and 10% filtered FBS. Cells were maintained in healthy conditions and were frequently subject to splitting and changing media when needed. All cells were kept in incubator at 37 °C with a supply of 5% carbon dioxide and 95% humidified environment.

### 2.4. Cell Seeding and Drug Treatments

A defined number of H1299 and A549 cells was seeded according to the experimental design. After seeding, cells were kept in the same sterilized growing conditions for 24 h to allow them to attain 20–30% confluence. Corresponding treatments were given to reach the optimal working concentrations and times as referred to in figure legends and results section. AdipoRon, 2-DG and Compound-C were suspended in DMSO, whereas Oxamate was suspended in distilled H_2_O. DMSO, dH_2_O or both (% *v*/*v*) were included as negative control in each experimental procedure. Two hours pretreatment was applied for all the procedures in which the usage of 2-DG, Oxamate and Compound-C was provided.

### 2.5. Cell Counting and Fixation

Cell proliferation analysis was implemented by counting cells under the microscope and comparing the relative number among differential experimental conditions. Briefly, 6 × 10^4^ cells were seeded in a 6-well plate and differentially treated as mentioned in the following section. Once completed, the cells were detached using trypsin and resuspended in fresh media. Cell counting was performed by thoroughly mixing cells plus media and loading the Bürker chamber in duplicates. Where required, media containing cells were subsequently centrifuged at 1200 rpm for 5 min. The supernatant was removed and the cell pellet was washed once in PBS before another centrifugation. Finally, the cell pellet was dissolved in 300 μL PBS plus 700 μL chilled absolute ethanol added drop by drop to fix the cells. The samples were preserved at −20 °C until the analysis.

### 2.6. Flow Cytometric Analysis

The cell population was differentiated into various phases of the cell cycle, employing Propidium Iodide (PI) as a DNA staining solution. Cells at 6 × 10^4^ density were seeded and treated in a dose-dependent manner using 6-well plates. After 24 h, the samples were fixed and stored at −20 °C until analysis. When ready, cells were first spun down at 1200 rpm for 5 min, and then suspended in a mixture of PI (15 μg/mL) and RNase A (10 μg/mL) dissolved in PBS. Once incubated at room temperature for 10 min in the dark, FACS-Celesta (BD Biosciences, Franklin Lakes, NJ, USA) was used to examine the cell cycle profile, recording the fluorescence intensity of differentiated DNA content. Cytofluorimetric analysis was made by estimating the percentage of the cell population in each of the subG1 (<2n), G0/G1 (2n), S (2n–4n) and G2/M (4n) phases of the cell cycle for at least 20,000 events [30].

### 2.7. Cell Viability Assessment

The mitochondrial activity of live viable cells was evaluated using MTT assay. Approximately 1.8 × 10^3^ cells were seeded in a 96-well plate. After 24 h, cells were subjected to respective drug treatments as stated in the figure legends and results section. At a predetermined time point, the experiment was stopped by adding 10% of 2,5-diphenyltetrazolium bromide (MTT) solution in each well, with cells being kept in an incubator for three more hours. When water-insoluble purple formazan crystals were formed, the media were removed carefully, and the purple crystals were dissolved in 10% Isopropanol-HCl solution. The optical density was detected at 570 nm using a Microplate Reader (Bio-Rad Laboratories, Hercules, CA, USA). Each condition was tested in replicates for every experimental assay.

### 2.8. Colony-Forming Assay

H1299 and A549 cells at a density of 1.5 × 10^3^ were seeded in 6-well plates. After 24 h of incubation, the cells were treated with an optimal concentration of AdipoRon, either alone or in combination with glycolytic inhibitors, along with their respective negative control. Colonies of H1299 cells were stopped after seven days, whereas A549 cells were stopped after nine days. At the end of the experiment, the plates were stained with crystal violet solution (1% aqueous solution) and the colonies were allowed to adhere the dye on their surface for 10 min. Thereafter, each well was washed with distilled H_2_O at least two times. After being dried at room temperature for 24 h, the colonies were first acquired in photographic way, and then dissolved in 10% acetic acid. Quantitative analysis was carried out using an Infinite 200 PRO Microplate Reader (Tecan Life Sciences, Männedorf, Switzerland), recording the absorbance at a 590 nm wavelength.

### 2.9. Media Collection and Analysis

Exhausted media were collected at the end of the respective experimental procedures. After being centrifuged at 1200 rpm for 5 min, the supernatant was picked up and analyzed for both the glucose and lactate amount using a Stat Profile Prime Plus instrument (Nova Biomedical, Waltham, MA, USA). The concentration of these two metabolites was also measured in fresh culture medium to define the effective consumption during incubation. Δ-glucose and Δ-lactate express the difference between the beginning and end of treatment in both glucose and lactate magnitude, respectively (the amount actually consumed by cells).

### 2.10. Cell Lysis and Protein Quantification

A number of 4 × 10^5^ cells were seeded into 100 mm Petri plates and subjected to the requested drug supplementation. Thereafter, cells were scraped manually, and centrifugation was executed at 1200 rpm for 5 min. The supernatant was removed and the pellet was stored immediately at −80 °C until the next processing phase. Cell lysis was carried out by dissolving the pellet in a buffer composed of protease/phosphatase inhibitors and RIPA. Cell lysis was executed by performing vortex three times with a 10 min interval and centrifuging at 12,000 rpm for 20 min. The supernatant was separated and subject to total protein quantification using the standard protocol of the Bradford Assay (39222; SERVA, Heidelberg Germany). Lastly, absorbance was estimated at 570 nm using UV/VIS Spectrophotometer V-550 (JASCO, Mary’s Court Easton, MD, USA). All the above-mentioned steps were made at a controlled temperature (4–6 °C cooling).

### 2.11. Western Blotting

The samples were prepared according to the following protocol: (i) mixing total protein with 2X Laemmli Buffer; (ii) vortexing the mixture for 10 s; (iii) heating for 6 min at 95 °C; (iv) spinning for 5 min at 6500 rpm. Sodium dodecyl sulfate–polyacrylamide gel electrophoresis (SDS-PAGE) was carried out to resolve the 20–30 μg of sample protein in 10–12% gel according to the molecular size of the targeted protein at 135 V. Thereafter, proteins from the gel were transferred to the nitrocellulose membrane (Amersham^TM^ Protran^TM^ Premium 0.45 µm) using wet transfer in a Mini Trans-Blot system (Bio-Rad Laboratories) at 100 V for 90 min. Ponceau staining solution was poured on the membrane and kept in a horizontal shaker for 6 min to ensure the quality of protein transfer. The membrane was washed three times with tris-buffered saline with 0.05% Tween^®^ 20 (TBS-T) for 5 min. Non-specific slots were blocked on film by treating with 5% non-fat dry milk for one hour. Then, films were washed and incubated with respective rabbit primary antibody. The overnight incubation with primary antibody was conducted in a cold room at 4 °C. The next day, the films were processed using TBS-T washing and incubation with conjugated Anti-Rabbit Horseradish peroxidase (HRP) for one hour. When the secondary antibody attached with the epitopes of primary antibody, the films were exposed to the Excellent-Chemiluminescent Substrate Kit (E-IR-R301; Elabsciences). The horseradish peroxidase enzyme of the secondary antibody catalyzed the substrate and produced a light emission that was captured using ChemiDoc™ (Bio-Rad Laboratories). Later, blots were analyzed by measuring the intensity of the targeted protein expression using Image J software (Version 1.52a) for quantitative analysis.

### 2.12. Statistical Analysis

All experimental data were analyzed statistically using GraphPad Prism Software (Version 8.0.2). The results were described as the Mean ± Standard Deviation (SD) of at least three replicates. Comparisons between the two groups were made using Welch’s t-test. An analysis of variance (ANOVA) was tested among more than two groups. Specifically, Brown–Forsythe and Welch ANOVA tests were carried out to estimate the mean difference between the pairs of groups. A *p*-value of less than 0.05 was considered as significant.

## 3. Results

### 3.1. ADIPOR1 and ADIPOR2 Display Ambiguous Results in Predicting NSCLC Survival

**Figure 1:**
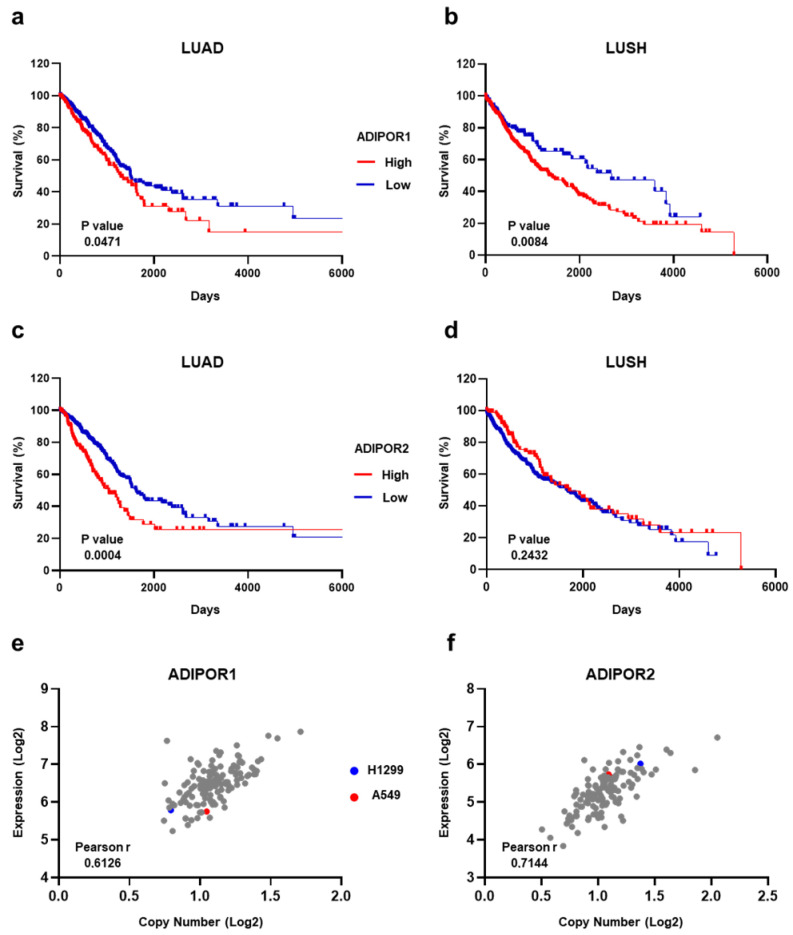
Estimation of ADIPOR1 and ADIPOR2 expression levels in both NSCLC patients and cell lines. (**a**) LUAD and (**b**) LUSH Kaplan–Meier survival curves obtained using 65.53 and 46.89 FPKM as a cut-off for ADIPOR1 expression levels, respectively. (**c**) LUAD and (**d**) LUSH Kaplan–Meier survival curves using 16.04 and 29.64 FPKM as ADIPOR2 cut-off, correspondingly. (**e**) ADIPOR1 and (**f**) ADIPOR2 plots showing correlation analysis between expression and copy number among NSCLC cells. Each dot recognizes a NSCLC cell line included in DepMap portal. *p* value by Log-rank (Mantel–Cox) test. Linear relationship between expression and copy number by Pearson’s correlation coefficient (Pearson r) test.

Before investigating the therapeutic potential of AdipoRon in NSCLC, we explored the chance of measuring ADIPOR1 and ADIPOR2 levels to predict overall survival. The NSCLC-related expression data of both ADIPOR1 and ADIPOR2 were extrapolated from the Human Protein Atlas (HPA) program, using The Cancer Genome Atlas (TCGA) datasets [27]. The row results were initially analyzed according to HPA guidelines, and then the Kaplan–Meier curves were plotted with the purpose of correlating ADIPOR1 and ADIPOR2 expression with NSCLC patient survival. Irrespective of the histological subtype, Figure 1a,b display a better outcome in terms of survival for NSCLC patients who exhibit lower ADIPOR1 levels. Conversely, having an elevated ADIPOR2 expression seems to provide long-term benefits in extending life expectancy (Figure 1c,d).

Moving to NSCLC cell lines, we inspected both the mRNA and copy number across ADIPOR1 and ADIPOR2 receptors. By exploiting the Cancer Dependency Portal (DepMap) [29], ADIPOR1 and ADIPOR2 statistics were obtained for all the available NSCLC cell lines, including H1299 and A549, that we chose as the representative models for this study. Whilst A549 is on the average for copy number value, both cell lines are just below the median concerning the ADIPOR1 expression levels (Figure 1e). With regard to ADIPOR2, A549 displays a copy number and mRNA amount quite close to the median, whereas the H1299 numbers are above the standard for both variables (Figure 1f).

Overall, the TCGA-related data suggest that ADIPOR1 and ADIPOR2 have an ambiguous meaning in predicting NSCLC survival; meanwhile, no major confounders exist in the H1299 and A549 cells for these genes.

### 3.2. AdipoRon Inhibits Viability, Growth and Colony Formation in NSCLC Cells

**Figure 2:**
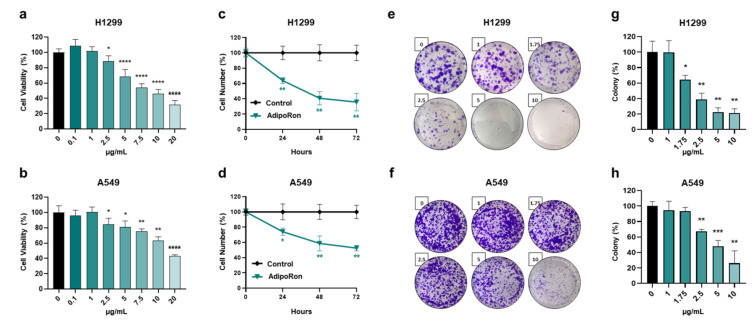
Evaluation of the AdipoRon-related effects on viability, growth and colony formation in NSCLC cell lines. (**a**) H1299 and (**b**) A549 cells undergo increasing concentrations of AdipoRon (from 0 to 20 µg/mL) for 72 h before being assessed for viability index using MTT assay. (**c**) H1299 and (**d**) A549 cells were supplemented with 10 µg/mL for a time span of approximately 72 h. Cell numbers were estimated in response to both AdipoRon and untreated counterpart (control) every 24 h. (**e**,**g**) H1299 and (**f**,**h**) A549 cells were examined for the clonogenic potential in presence of growing amount of AdipoRon (from 0 to 10 µg/mL) after 7 and 9 days, respectively. Representative pictures of crystal violet-stained colonies are shown in panels (**e**,**f**), while quantification analysis from multiple independent experiments is stated in panels (**g**,**h**). All the reported tests were replicated at least three times (n ≥ 3). Mean value ± SD were plotted on graph in % of control. * *p* < 0.05, ** *p* < 0.01, *** *p* < 0.001, **** *p* < 0.0001 using Welch’s *t*-test.

According to the previously published studies [20,21,22,23,31,32], we initially exposed H1299 and A549 cells to escalating doses of AdipoRon (from 0 to 20 µg/mL) for 72 h. Thereafter, the activity of nicotinamide adenine dinucleotide phosphate (NADPH)-dependent cellular oxidoreductase enzymes was assessed as an indirect estimation of the cell viability. Figure 2a,b markedly prove AdipoRon’s effectiveness in mitigating NSCLC viability. Dose–response experiments also provided additional details about the different AdipoRon sensitivity between H1299 and A549 cells. Quite representative of this are the results achieved in response to 10 µg/mL AdipoRon, where we recorded greater susceptibility in H1299 (54% inhibition) as compared with A549 (~36.5%).

To further corroborate the AdipoRon-mediated antiproliferative features detected in NSCLC cells, we subsequently performed time course experiments aimed at addressing potential changes in growth rate. Selecting 10 µg/mL as the effective working dosage in H1299, we observed a count decline of 36%, 59% and 64% after 24, 48 and 72 h of AdipoRon administration, respectively (Figure 2c). A similar but less marked trend was obtained in A549, where AdipoRon provoked a cell growth impairment of approximately 47% at 72 h as the best performance (Figure 2d). Analogous experiments carried out using 5 µg/mL AdipoRon confirmed H1299’s ability to respond more effectively to AdipoRon (Figure S1).

Given that clonogenic assay is widely accepted as a suitable test for evaluating undifferentiated potential and anchorage-independent growth in cancer research [33], we successively addressed the AdipoRon impact on NSCLC stemness. Therefore, H1299 and A549 cells were seeded at an extremely low confluence, before being treated with increasing AdipoRon concentrations (from 0 to 10 µg/mL). As reported in Figure 2e,g, AdipoRon dramatically reduced H1299 clonogenic potential, affecting both the size and number of the newly formed colonies, even in small concentrations like 1.75 µg/mL. Consistent results were also obtained in A549 cells where the minimal effective dose of AdipoRon was 2.50 µg/mL (Figure 2f,h).

Taken together, these findings indicate a stronger and deeper AdipoRon-mediated outcome in limiting viability, growth and colony formation in NSCLC cell models.

### 3.3. AdipoRon Induces Cell Cycle Retardation in NSCLC Cells

To figure out whether the AdipoRon-mediated anticancer effects were borne out by changes in cell cycle progression, we successively quantified the cellular DNA amount using Propidium Iodide (PI) as the base pairs intercalating dye. Hence, H1299 and A549 were treated for 24 h with three effective dosages (5, 10 and 20 µg/mL) of AdipoRon, and the cell cycle distribution was assessed using flow cytometry analysis.

**Figure 3:**
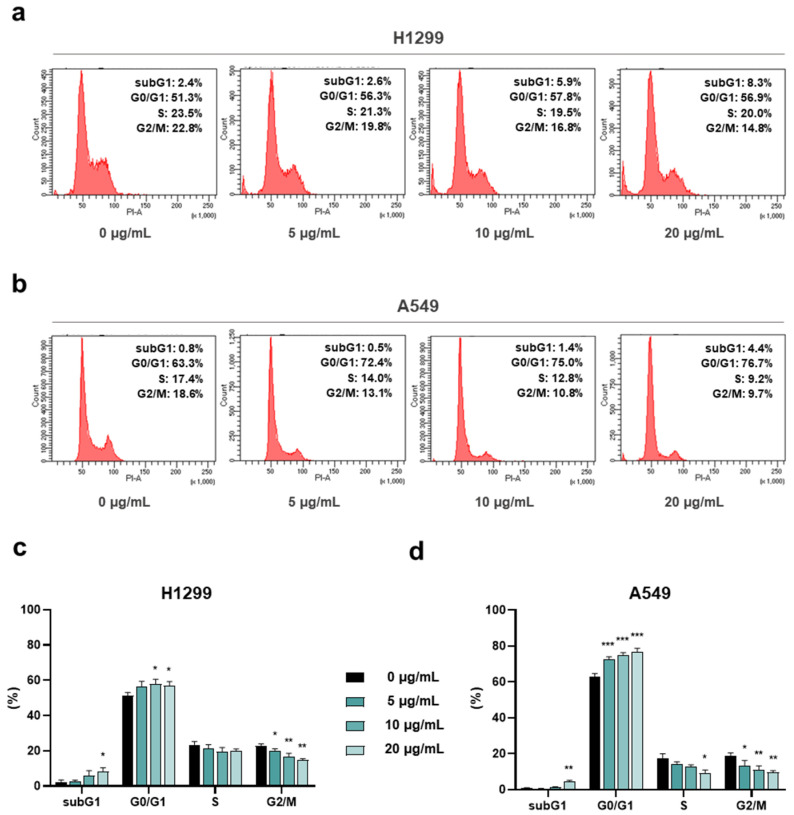
Assessment of the AdipoRon-mediated outcome on cell cycle distribution in NSCLC cell lines. H1299 and A549 cells were scrutinized for the assignment into the different cell cycle stages as a result of 24 h escalating AdipoRon doses (0, 5, 10 and 20 µg/mL). Panels (**a**,**b**) show typical histogram plots achieved in both H1299 and A549 cells, respectively. Statistical analysis of at least three independent experiments is reported in panels (**c**) for H1299 and (**d**) for A549. Data are represented in percentage as average value ± SD. * *p* < 0.05, ** *p* < 0.01, *** *p* < 0.001, using Welch’s *t*-test.

Nuclear DNA staining revealed an AdipoR aptitude in increasing G0/G1 at the expense of the G2/M phase in both NCSLC cells (Figure 3a,b). Nevertheless, whilst the magnitude of G0/G1 increase was quite constant across the different concentrations tested in H1299 (6% intensification compared to the untreated counterpart), a clearer dose–response was detected in A549 (Figure 3c,d). In this latter NSCLC model, the amount of non-dividing cells raised by approximately 9%, 11% and 14% in response to 5, 10 and 20 µg/mL AdipoRon, correspondingly (Figure 3b,d). An opposite but similar G2/M trend was observed in both NSCLC cells, where reduction values of about 8% were detected in reaction to the highest AdipoRon concentration (Figure 3a–d).

Altogether, this evidence further recognizes AdipoRon as an antiproliferative compound in NSCLC and supports its peculiarity in slowing down cell cycle progression.

### 3.4. AdipoRon Provokes Glucose Consumption and Lactate Accumulation in NSCLC Cells

Starting from a simple visual observation, we noticed that the AdipoRon-containing media were more yellowish when the experiment ended. This phenomenon was quite consistent in H1299, where RPMI1640 made the color change clearer than DMEM.

Phenol red is widely accepted as a visual pH indicator dye for in vitro cell culture [34,35]. Waste products and overgrowth usually are the main culprits for media color variation. But since the number of cells was higher in the untreated cells (control), where no changes were detected in media dye, we speculated that AdipoRon could affect NSCLC cellular waste. As a final product of anaerobic glycolysis, lactate is considered as one of the key catabolites responsible for media acidification and its toning towards yellow-orange shade [36].

**Figure 4:**
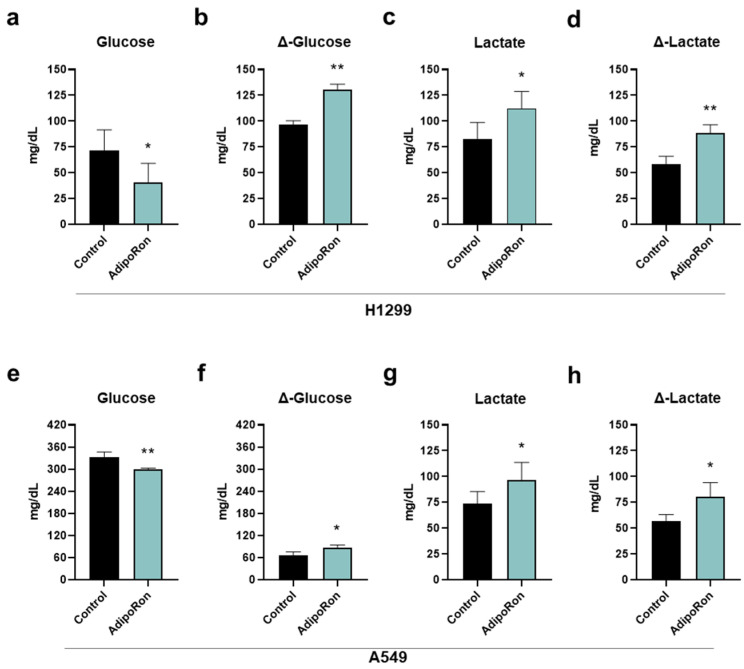
Determination of glucose and lactate medium contents in AdipoRon-treated NSCLC cell lines. H1299 and A549 cells were treated and not (control) with 10 µg/mL AdipoRon for 48 h. Upon completing the experimental procedure, culture medium was collected and subsequently analyzed for both glucose and lactate amount. (**a**) Glucose, (**b**) Δ-glucose, (**c**) lactate, (**d**) Δ-lactate in H1299. (**e**) Glucose, (**f**) Δ-glucose, (**g**) lactate, (**h**) Δ-lactate in A549. Results were graphically represented as mean ± SD of not less than three distinct experiments. * *p* < 0.05, ** *p* < 0.01 using Welch’s *t*-test.

In the light of this emerging evidence, we measured both glucose and lactate concentration in medium containing and medium without (control) 10 µg/mL AdipoRon after 48 h of treatment. Figure 4a,e display a lower presence of glucose in either H1299 or A549 AdipoRon-treated media, thus suggesting an enlarged consumption by these cells (Figure 4b,f). Specifically, the glucose amount dropped by 45% and 11% after 48 h of AdipoRon treatment in H1299 and A549, respectively (Figure 4a,e). In the same culture broth, we also recorded a higher lactate content as a result of AdipoRon administration (Figure 4c,g), assuming a greater accumulation in the presence of this compound (Figure 4d,h). The levels of lactate raised by almost 35% as a consequence of the AdipoRon outcome in both NSCLC cells (Figure 4c,d,g,h).

Collectively, these data prove that AdipoRon modulates glucose consumption and lactate accumulation in NSCLC.

### 3.5. Glycolysis Impairment Enhances AdipoRon-Mediated Antiproliferative Action in NSCLC Cells

Assuming the augmented intake of glucose and the buildup of extracellular lactate as a direct effect of the AdipoRon treatment in NSCLC, we later investigated the role of both glycolysis and lactic fermentation on its antiproliferative features.

**Figure 5:**
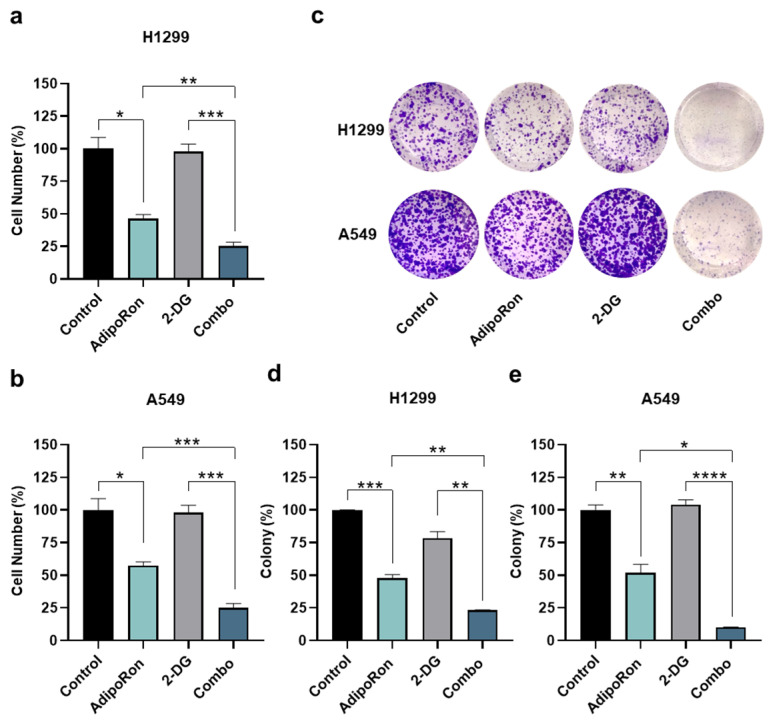
Investigation of the AdipoRon plus 2-Deoxy-Glucose consequences on growth and colony formation in NSCLC cell lines. (**a**) H1299 and (**b**) A549 cells were incubated with 10 µg/mL AdipoRon and 0.5 mM of 2-Deaoxy-Glucose (2-DG) for 48 h, both as single drug and together. Thereafter, cell numbers were estimated and interpolated on graphs as % of control. (**c**,**d**) H1299 cells were exposed to 2.5 µg/mL AdipoRon, 0.5 mM 2-DG and AdipoRon plus 2-DG for 7 days before staining newly formed colonies with crystal violet. (**c**,**e**) A different AdipoRon dosage (5 µg/mL) and time (9 days) were employed for A549 colony assay. (**c**) Representative results achieved in H1299 and A549. (**d**,**e**) Statistical analysis performed on multiple colony tests in H1299 and A549 cells, respectively. All the stated experiments were replicated at least three times (n ≥ 3). Median value ± SD were plotted on graph in % of control. * *p* < 0.05, ** *p* < 0.01, *** *p* < 0.001, **** *p* < 0.0001 using Brown–Forsythe and Welch ANOVA tests.

To this end, H1299 and A549 cells were initially exposed to 10 µg/mL AdipoRon for 48 h, both in the presence and in the absence of the glycolytic inhibitor 2-Deoxy-D-glucose (2-DG) [37]. As largely expected, AdipoRon-treated H1299 exhibited a cell proliferation decrease of over 50% after 48 h (Figure 5a). Strikingly, a combination of AdipoRon plus 2-DG (Combo) further declines cell growth, reaching an inhibition index of 78% (Figure 5a). Comparable results were also achieved in A549, where an additional 20% decline was detected in Combo compared with a single treatment of AdipoRon (Figure 5b). Although 2-DG alone had no impact in terms of cell number reduction, it effectively prevented both glucose consumption and lactate release in these cells (Figure S2). Colony-forming assays corroborated the best efficacy of Combo than AdipoRon, with an inhibition degree similar to those achieved in counting results (Figure 5c–e).

**Figure 6:**
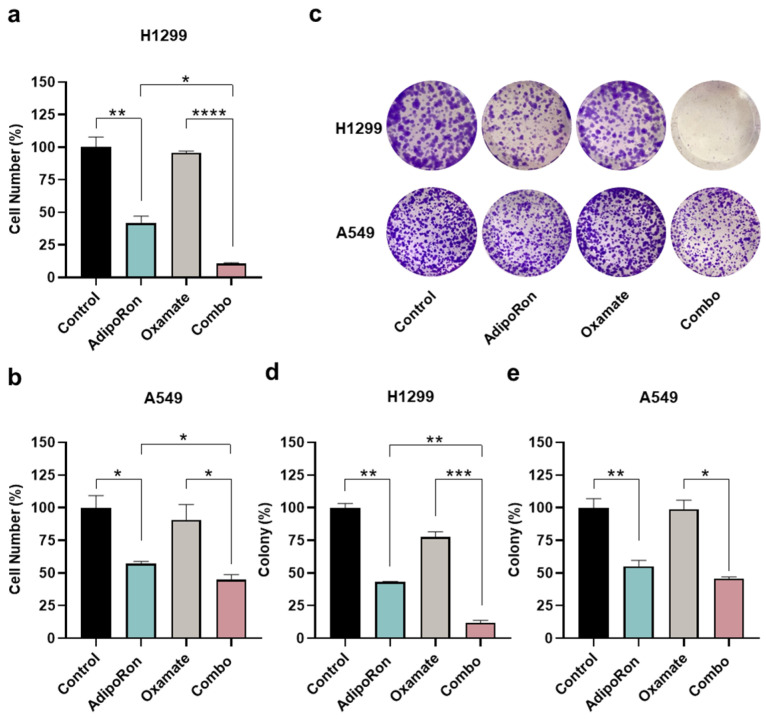
Examination of the AdipoRon plus Oxamate influences on growth and colony formation in NSCLC cell lines. (**a**) H1299 cells were exposed to 10 µg/mL AdipoRon and 1 mM of Oxamate for 48 h, either alone or in combination, before being inspected for the number of cells. (**b**) A549 results obtained in response to both single and double treatment with 10 µg/mL AdipoRon and 10 mM of Oxamate for 48 h. Quantitative analysis of colony-forming assays obtained in H1299 (**d**) and A549 (**e**) cells undergo the same treatments as defined in (**a**,**b**) for Oxamate after 7 and 9 days, respectively. An amount of 2.5 (H1299) and 5.0 (A549) µg/mL AdipoRon was used for assessing NSCLC stemness. (**c**) Typical colony images gained in H1299 and A549 cells as a result of different stimulations. Experimental procedures were replicated three times and displayed as average ± SD in % of control. * *p* < 0.05, ** *p* < 0.01, *** *p* < 0.001, **** *p* < 0.0001 using Brown–Forsythe and Welch ANOVA tests.

To address the lactic fermentation involvement instead, we employed Oxamate as a pyruvate analog and competitive inhibitor of lactate dehydrogenase (LDH) enzyme [38,39]. NSCLC cells were therefore treated with AdipoRon and Oxamate, either alone or in combination (Combo), with the purpose of addressing the relative outcome at 48 h. Figure 6a displays an even more dramatic impact by combining AdipoRon with Oxamate, expressly in H1299, where a declining number of almost 90% was recorded. Although A549 cells presented a lower susceptibility to Oxamate as a whole, the inhibition rate was 10% higher in Combo than AdipoRon (Figure 6b). A decrease in media lactate levels was used as evidence of LDH inhibition by Oxamate in NSCLC cells (Figure S3). Similarly to 2-DG, the assessment of clonogenic potential validated the Combo ability in impairing NSCLC stemness deeper than AdipoRon (Figure 6c–e).

Altogether, these results trace the possibility of combining AdipoRon with interfering glycolytic agents to facilitate the agonistic anticancer features in NSCLC.

### 3.6. AdipoRon Activates AMP-Activated Protein Kinase Alpha in NSCLC Cells

Among the intracellular signaling reported to be modulated by AdipoRon, we subsequently explored its potential consequences on AMPKα as one of the leading pathways involved in both NSCLC tumorigenesis and metabolic reprogramming [40,41]. Thus, H1299 and A549 were supplemented with 10 µg/mL AdipoRon for up to 72 h, before being inspected for the activation levels of AMPKα.

**Figure 7:**
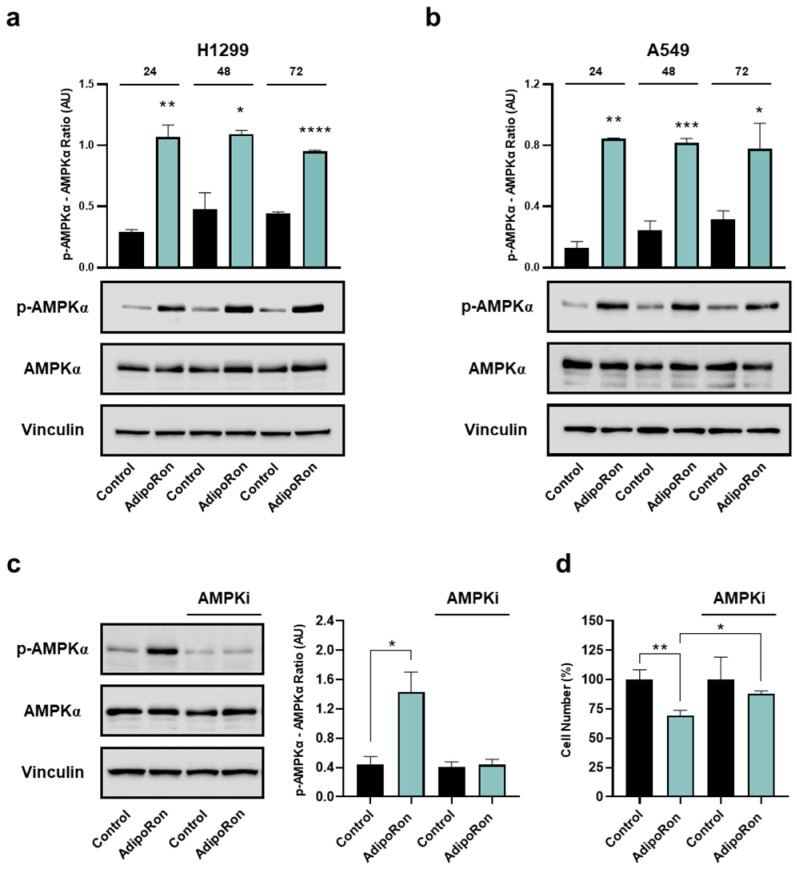
Determination of AMPKα involvement in the AdipoRon-mediated effects in NSCLC cell lines. H1299 and A549 were exposed to 10 µg/mL AdipoRon for 72 h with the purpose of defining AMPKα activation levels. Phospho AMPKα (Thr172), total AMPKα, Vinculin in H1299 (**a**) and A549 (**b**) cells. A549 cells were exposed to 10 µg/mL AdipoRon for 24 h, both in presence and absence of 5 µM Compound-C. Later, phospho AMPKα (Thr172), total AMPKα, Vinculin were assessed (**c**), together with the relative cell growth impact (**d**). Uncropped Western blotting films were reported in Figures S4 and S5. Arbitrary Unit (AU). * *p* < 0.05, ** *p* < 0.01, *** *p* < 0.001, **** *p* < 0.0001 using Brown–Forsythe and Welch ANOVA tests.

As reported in Figure 7a,b, AdipoRon strongly induced a massive phosphorylation of AMPKα at each time point analyzed in both NSCLC cells. Considering the non-significant changes in the total AMPKα protein amount, the increased phosphorylation triggers enhanced pathway activation as a result of AdipoRon administration.

To corroborate the AMPKα involvement in the AdipoRon-mediated growth effects, we also tested the consequences of the ATP-competitive AMPK inhibitor Compound-C on NSCLC proliferation. For this purpose, A549 cells were supplemented with AdipoRon, both in the presence and absence of Compound-C, before being analyzed for the relative growth rate at 24 h. Using 5 µM as an effective dosage in preventing AdipoRon-induced AMPKα phosphorylation (Figure 7c), the concomitant presence of both Compound-C and AdipoRon partially hampered the effectiveness of this latter, constraining the inhibition rate from 31% to 16% (Figure 7d).

Overall, the above data suggest that AdipoRon stimulates AMPKα phosphorylation, which is required to promote the NSCLC-mediated cell growth inhibition.

## 4. Discussion

Despite the growing number of both diagnostic and therapeutic tools for managing lung cancer, the death rate has decreased only marginally since 2014 [42]. Last year, an estimated 238,340 cases and 127,070 fatalities occurred in the United States, making lung malignancy the second-most commonly diagnosed cancer and the leading cause of cancer death for men and women worldwide [43]. Behind these impressive numbers, NSCLC represents the greatest proportion of care, since it accounts for almost 80% of all new lung cancer diagnoses [3]. The appropriate treatment for NSCLC is currently built on a multidisciplinary approach, which takes into consideration both the stage of disease and genetic signatures [44]. Pharmacologically, conventional with targeted and immune therapies constitute a milestone for NSCLC therapy [45,46,47]. However, due to both the failure of early detection and limitations of therapeutic strategies, the existing medications are only partially exhaustive for handling NSCLC, and thus additional options are absolutely required [48].

As in many other cancers, the fat-derived hormone adiponectin has been investigated in NSCLC, obtaining some conflicting data, especially for its diagnostic and prognostic significance [49,50]. Findings that support its therapeutic role seem more convincing in this tumor type. In this regard, we previously demonstrated that adiponectin has a significant effect in reducing A549 tumor cell proliferation by altering cell cycle progression and inhibiting CREB phosphorylation [51]. More recently, adiponectin has been reported to suppress both migration and invasion in NSCLC cells [52]. Irrespective of its impact in cancer models, the heavy molecular mass and the reduced half-life/stability of adiponectin has left no room for any therapeutic usage.

The synthetic adiponectin receptor agonist AdipoRon is emerging as a promising anticancer compound in different tumor types, including myeloma, breast, prostate and ovarian cancer [19]. In this respect, our group further contributed insights regarding AdipoRon antiproliferative features, with special reference to osteosarcoma and PDAC [20,23]. Aimed at addressing the AdipoRon candidacy in NSCLC treatment, herein we investigated its potential outcome in H1299 and A549 cells.

Albeit only initial, the study results reveal a remarkable AdipoRon-mediated outcome in limiting the viability, growth and stemness in NSCLC cells. As an additional proof of the AdipoRon antiproliferative features, we also detected a slowdown in cell cycle progression, consisting of an increased G0/G1 phase at the expense of G2/M. Remarkably, in five out of eight cancer types, in which AdipoRon has been proposed as antitumor molecule, the accumulation of a non-dividing state has been recorded, thus suggesting a precise mechanism of action for this compound [19]. Conversely, the cytotoxic effect does not seem to be the primary AdipoRon outcome in NSCLC, since just a minimal rise in debris (subG1 phase) has been noticed. A lack of cell death induced by AdipoRon is at odds with other cancers, such as PDAC, ovarian, leukemia and hepatocellular carcinoma [21,31,32,53]. However, it should be mentioned that nearly the totality of performed experiments for this study employ 10 ug/mL AdipoRon, which is quite a low concentration compared with previous ones. Therefore, we should not exclude the fact that increasing AdipoRon dosage could lead to cytotoxicity in NSCLC too.

Along with the biological outcome, we also observed a metabolic switching as a result of AdipoRon administration in NSCLC cells. AdipoRon supplementation caused higher glucose consumption and lactate accumulation in culture media. The relationship between cell cycle and cell metabolism is bidirectional and allows mutual regulation [54]. This close correlation is also experienced in our investigative models, given that interfering with the AdipoRon-induced glycolytic dependence greatly enhanced its antiproliferative peculiarities. Two additional considerations should be taken into account with regard to the metabolic reprogramming observed in reaction to AdipoRon. (i) Considering glycolysis as a well-known modulator of both tumor initiation and progression, a raised glucose intake may suggest a higher grade of malignancy [55]. Despite this basic association being factual in oncology, cancer and non-cancer cells could employ glucose for other purposes besides proliferation, such as replacing damaged macromolecules/organelles, producing glutathione to confer protection from free radicals and more [56]. Lemons and colleagues provided evidence about the high metabolic activity of quiescent fibroblasts compared with proliferating ones [57]. Similarly, hematopoietic stem cells prefer glycolysis to oxidative phosphorylation with the purpose of keeping a quiescent status [58]. (ii) AdipoRon-mediated glycolysis induction has recently been reported in PDAC by Manley et al. [59]. They elegantly proved that AdipoRon induces mitochondrial uncoupling and increased glucose uptake and utilization. Besides being in line with our current achievements, these findings may also provide additional links to other metabolic pathways. In this respect, we are currently investigating mitochondrial damage as a result of AdipoRon treatment in NSCLC. It is clear that glycolysis serves NSCLC cells to survive, since its impairment enhances AdipoRon efficacy. Different studies have shown the benefit of targeting enhanced glycolysis in cancer cells [60,61]. However, although numerous compounds have been developed and tested in pre-clinical and clinical stages, there is still a lack of effective strategies to target glycolysis-addicted cancer cells. AdipoRon-treated NSCLC cells could represent the suitable subset due to their high susceptibility to this metabolic route.

Although we are aware of the initial assessment of the signaling pathways, we highlighted a robust and persistent AMPK activation after AdipoRon exposure in NSCLC cells. While AMPK stimulation could suggest that AdipoRon is acting through the adiponectin axis, its involvement could also explain changes in both cell cycle progression and metabolism. Not surprisingly, AMPK activators have been reported to inhibit proliferation through G1/S arrest in different cancers, including NSCLC [62,63,64]. At the same time, AMPK affects glucose membrane transporters, thus promoting its uptake and the consequent lactic fermentation [65,66].

Nevertheless, as supported by the partial rescue observed in the presence of the AMPK inhibitor, it is likely that other molecular interactors are involved in the AdipoRon-mediated NSCLC outcome. In this respect, supplementary investigations are needed to better characterize the MAPK engagement that, as stated in different studies, could play a crucial role in mediating the AdipoRon efficacy [20,21,23]. PI3K-AKT-mTOR is another relevant pathway that deserves particular attention, since its modulation has also been documented in response to AdipoRon [21,67,68]. Akimoto and co-workers found an AdipoRon-induced phosphorylation of AKT in PDAC models on par with AMPK [21]. Conversely, KGN ovarian carcinoma of granulosa cells exhibited a decrease in AKT activation after AdipoRon exposure, which was associated with higher levels of phosphatase PTEN [68].

Lastly, the fast kinetics of all these mentioned pathways opens up to additional remarks, such as the assessment of potential short-term activations which could provide a more complete overview of the signaling network, such as in discriminating primary (initial) from secondary (late) responses [20,21,67,69].

## 5. Conclusions

In this study, we first emphasized the adiponectin receptor agonist AdipoRon as a potential anticancer molecule in NSCLC. Besides impairing viability, proliferation and clonogenic potential, we also observed an AdipoRon-induced adaptive glycolytic dependence. Mechanistically, AMPK phosphorylation sustained the antiproliferative outcome, since its ablation significantly counteracted the AdipoRon effectiveness.

Together with the existing results, these findings encourage the design of a future investigation aimed at exploring AdipoRon employment as a candidate in cancer and NSCLC therapy.

## Data Availability

The datasets used and/or analyzed during the current study are available from the corresponding author on reasonable request.

## Supplementary Materials

The following supporting information can be downloaded at: https://www.mdpi.com/article/10.3390/cancers16152633/s1, Figure S1: Growth curves obtained in reaction to 5 µg/mL in NSCLC cell lines; Figure S2: Residual glucose and lactate amount after 2-DG treatments in NSCLC cell lines; Figure S3: Residual glucose and lactate amount after Oxamate treatments in NSCLC cell lines; Figure S4: Uncropped Western blotting films reported in Figure 7 panel (**a**) and (**b**); Figure S5: Uncropped Western blotting films displayed in Figure 7 panel (**c**).
