# Supplementary material for: Adiponectin Receptor Agonist AdipoRon Inhibits Proliferation and Drives Glycolytic Dependence in Non-Small-Cell Lung Cancer Cells
Source: Cancers (Basel). 2024 Jul 24;16(15):2633. doi: 10.3390/cancers16152633 (PMC11312309; doi:10.3390/cancers16152633)
Supplement: Supplementary file 1 [file cancers-16-02633-s001.zip › cancers-3081572-supplementary.pdf]

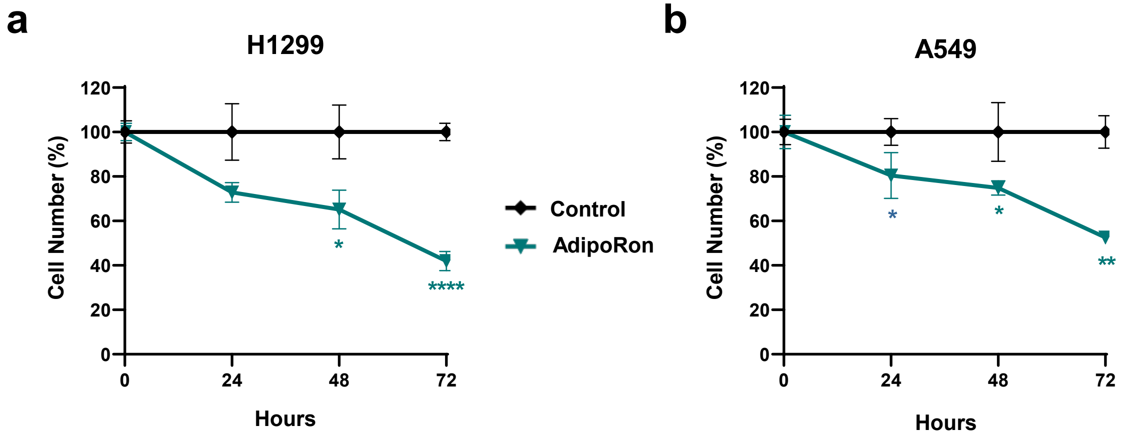

Figure S1. Growth curves obtained in reaction to 5 µg/mL in NSCLC cell lines. **(a)** H1299 and **(b)** A549 cells were supplemented and not (control) with 5 µg/mL for up to 72 hours. *p < .05, **p < .01, ****p < .0001 by Welch's t-test.

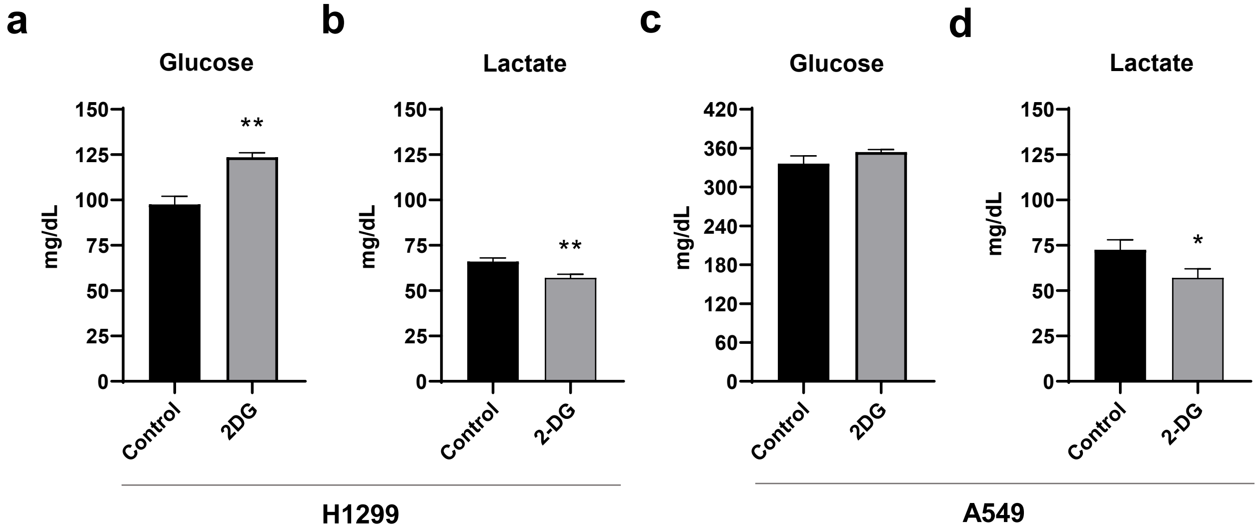

Figure S2. Residual glucose and lactate amount after 2-DG treatment in NSCLC cell lines. Remaining glucose (**a,c**) and lactate (**b,d**) in culture media after 48 hours exposure to 0.5 mM 2-DG in H1299 and A549 cells. *p < .05, **p < .01, by Welch's t-test.

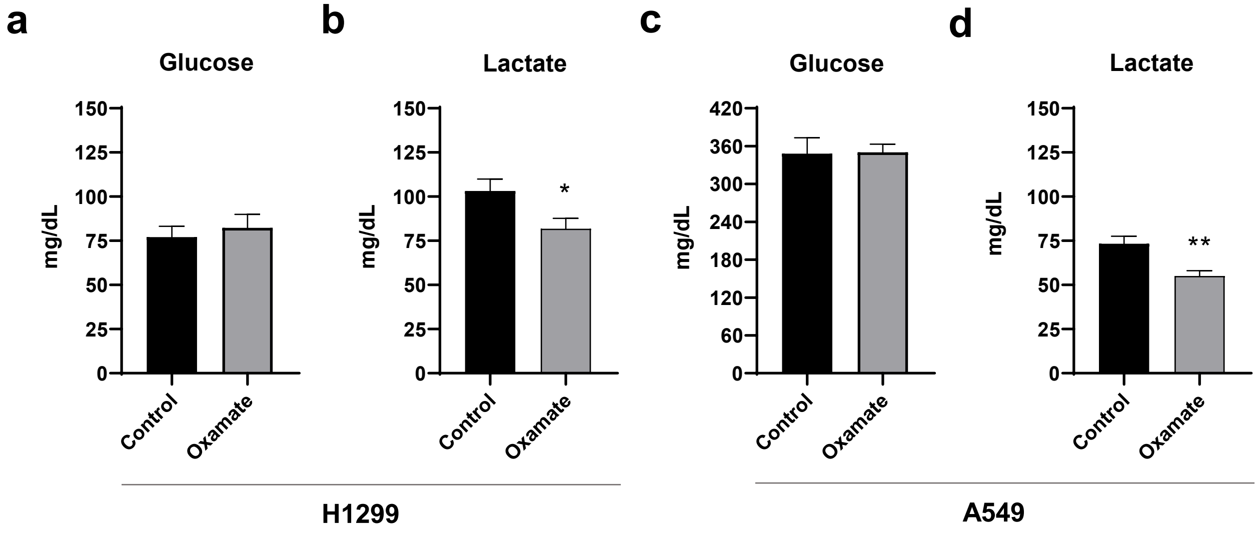

Figure S3. Residual glucose and lactate amount after Oxamate treatment in NSCLC cell lines. Remaining glucose (**a,c**) and lactate (**b,d**) in culture media after 48 hours exposure to 1 and 10 mM Oxamate in H1299 and A549 cells, respectively. *p < .05, **p < .01, by Welch's t-test.

a

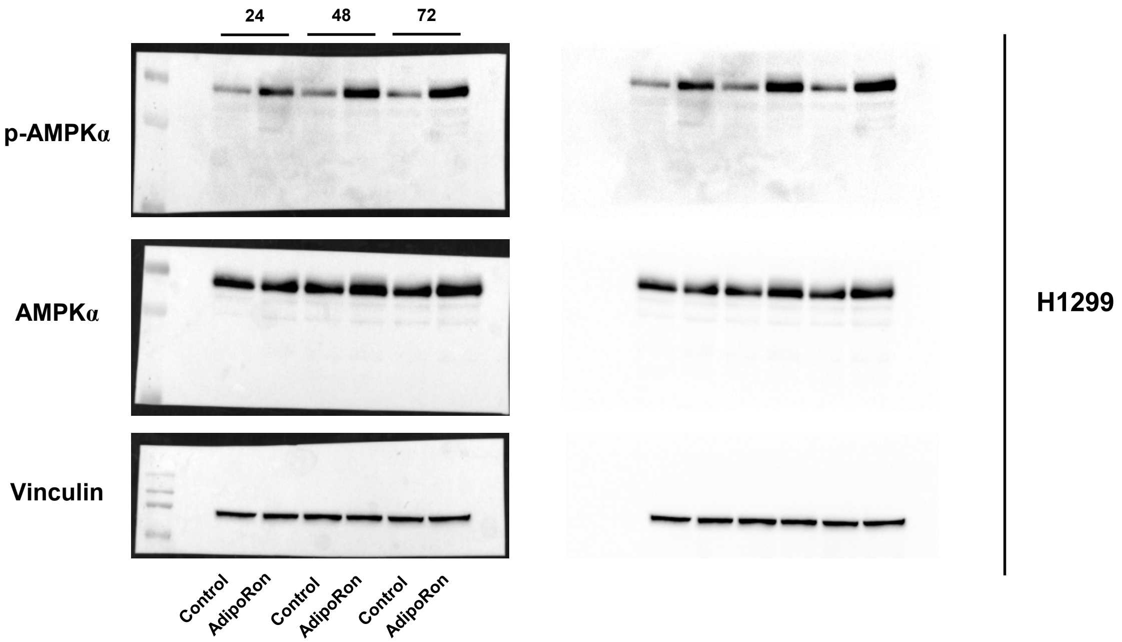

b

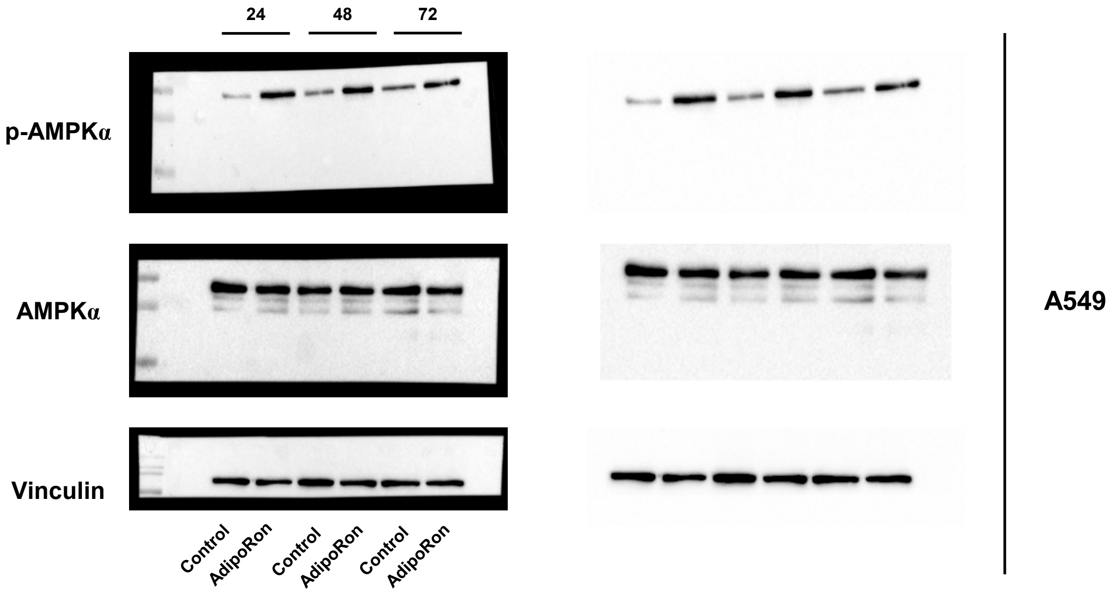

Figure S4. Uncropped Western blotting films reported in Figure 7 panel (**a**) and (**b**).

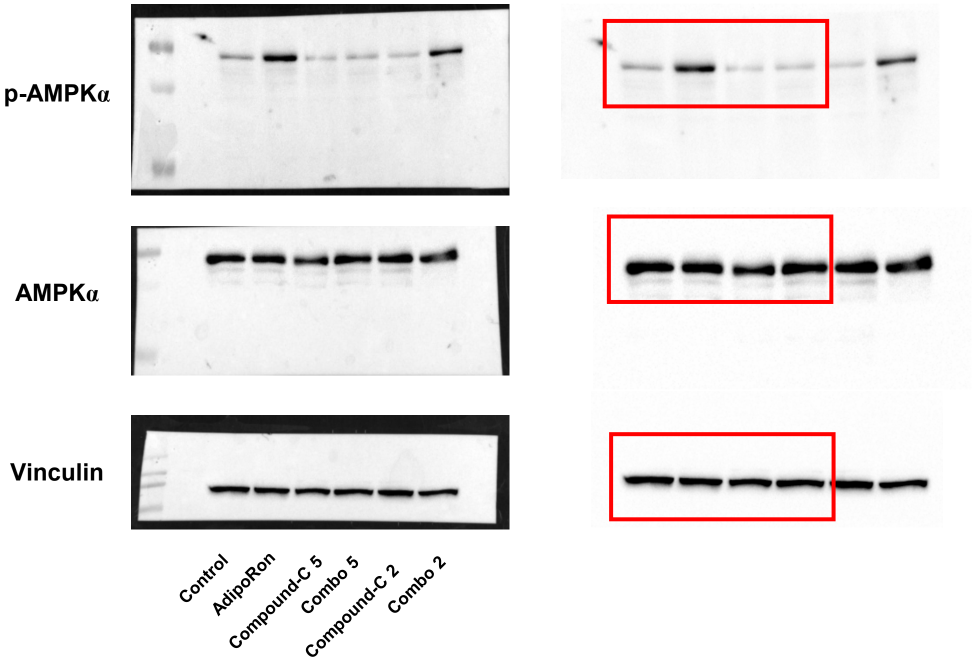

Figure S5. Uncropped Western blotting films reported in Figure 7 panel (**c**).
